# Mobile-assisted vocabulary learning through the Shanbay App outside the classroom: Effects of self-regulation and peer scaffolding

**DOI:** 10.3389/fpsyg.2022.993224

**Published:** 2022-10-06

**Authors:** Fengping Guo, Yuhan Zhang, Zhixin Wu

**Affiliations:** Department of Foreign Languages, The Southeast University, Nanjing, China

**Keywords:** mobile-assisted vocabulary learning, self-regulation, peer scaffolding, number of days, sum of words

## Abstract

Recent decades have witnessed an increasing academic interest in mobile-assisted vocabulary learning. To explore the possible influencing factors on learning outcomes, this study aimed at examining the effects of self-regulation and peer scaffolding on mobile-assisted vocabulary learning among undergraduate students using Shanbay App beyond the classroom. To this end, altogether 71 intermediate-level English learners aged 17–19 years were chosen as participants, with 37 in the experimental group (with peer scaffolding) and 34 in the control group (without peer scaffolding). Data were collected through the Shanbay App regarding participants’ vocabulary learning performance, a self-regulation questionnaire and semi-structured interviews. The results of factorial ANOVA revealed that peer scaffolding significantly affected mobile vocabulary learning in terms of the days spent in vocabulary learning and the sum of words participants have learned; a main effect of self-regulation and an interaction effect of self-regulation and peer scaffolding were also observed on the sum of learned words. The research is innovative in providing a motivational peer scaffolding framework in mobile vocabulary learning settings, and may provide pedagogical implications for vocabulary teaching in EFL context in higher education.

## Introduction

Mobile-assisted language learning (MALL) has recently become a popular research area in the SLA field. It has been considered to facilitate language learning effectively by providing a contextually sensitive, socially connective and personalized mobile-mediated learning environment (Lin and Lin, 2019). To investigate whether mobile vocabulary learning outweigh the traditional paper-based learning, or the effectiveness of mobile vocabulary learning outcomes, a great amount of research has been conducted, among which the predominant approaches include using mobile applications (apps), short message services (SMS), and multimedia message service (MMS) (Derakhshan and Kaivanpanah, 2011; Hsu and Lee, 2011; Sandberg et al., 2014; Suwantarathip and Orawiwatnakul, 2015; Kohnke, 2020).

As noted in previous research, to guarantee the effectiveness of mobile vocabulary learning, personal factors like motivation and skills in self-regulated learning (SRL) activities played an indispensable role in online learning settings (Viberg and Andersson, 2019). However, numerous studies have focused on using mobile technology in language learning classroom, yet using it in self-initiated and SRL beyond the classroom remains to be further explored (Lai et al., 2022).

In addition, previous literature has also highlighted the need to improve Students’ SRL through outside support like peer scaffolding in technology-enhanced learning environments (Shyr and Chen, 2018). However, most of them focused on how cognitive or meta-cognitive peer scaffolding may influence mobile learning outcomes (Wu et al., 2012; Lan, 2013; Kuo et al., 2014; Rashid et al., 2016; Rasheed et al., 2021), yet whether motivational peer scaffolding could equally scaffold Students’ learning has rarely been examined (Mi et al., 2020).

To explore this issue, the current study is aimed at investigating the effects of self-regulation and motivational peer scaffolding on mobile-assisted vocabulary learning through the Shanbay App, and is expected to shed light on the current SRL and MALL research by incorporating a peer scaffolding framework, and also on the improvement of college English vocabulary teaching through the application of mobile devices.

## Literature review

### Mobile-assisted vocabulary learning

Vocabulary learning has long been known as one of the major challenges for L2 learners, and the anywhere and anytime features of mobile technologies made it possible to digest the great amount of words within short periods of time (Sung et al., 2016), and take charge of their development at their own pace (Norris et al., 2011; Hung et al., 2012). These characteristics with L2 vocabulary learning has made it the most frequently focused one in the MALL field (Darmi and Albion, 2014; Burston, 2015). As noted by Lin and Lin (2019), in general, the mobile-assisted vocabulary learning can be roughly divided into two strands: (a) the effects of short message delivery (e.g., SMS, MMS) and reception methods on L2 word learning (Derakhshan and Kaivanpanah, 2011; Zhang et al., 2011; Suwantarathip and Orawiwatnakul, 2015) and (b) the effects of personalized, tutorial mobile learning apps (Sandberg et al., 2014; Rachels and Rockinson-Szapkiw, 2018) or context-aware mobile technologies on L2 word learning (Chen and Chung, 2008; Wu, 2014, 2015a,b; Ono et al., 2015).

Previous studies have shown the facilitating effects of mobile message delivery on L2 learners’ target word learning through receiving and sending feedback between partner and teachers (Derakhshan and Kaivanpanah, 2011; Zhang et al., 2011; Suwantarathip and Orawiwatnakul, 2015), and have also revealed that using mobile word-learning applications could help improve word retention results as well as enhance learners’ learning interest through word games, flashcard reinforcements, and well-designed online language learning programs (Basoglu and Akdemir, 2010; Hsu and Lee, 2011; Sandberg et al., 2014; Alenezi et al., 2022). In addition, learners’ self-awareness (Liu et al., 2008) or self-regulation was also found to increase through vocabulary learning with mobile devices (Kondo et al., 2012; Lei et al., 2022). However, the use of mobile learning Apps inside and outside the classroom may bring about different learning results (Kohnke, 2020). Despite the facilitating role of mobile Apps in fostering learning independence, research probing into learners’ use of Apps outside the classroom is not yet sufficient (Burston, 2015; Stockwell and Liu, 2015; Lai et al., 2022).

Furthermore, though the effectiveness of mobile vocabulary learning has been well justified in previous research, there were still some research reporting no positive correlations between mobile-assisted vocabulary learning approach and the learning results (Stockwell, 2010; Derakhshan and Kaivanpanah, 2011). For instance, some studies presented that learners tended to treat mobile devices as social apps rather than word learning tools (Stockwell, 2010), and may even find mobile vocabulary learning distracting, as they may encounter technical problems and find it difficult to concentrate on word learning (Lu, 2008). To summarize, the above research results pose a challenge to the effect of mobile-assisted vocabulary learning (Lin and Lin, 2019).

### Self-regulated learning

As an important factor in autonomous learning, SRL can be understood as “the process where learners activate and sustain cognitions which are systematically oriented toward attaining certain goals” (Schunk and Zimmerman, 1994). In out-of-class online learning settings, students were obliged to regulate and manage their own learning activities and tasks independent of their teachers (Rasheed et al., 2020), and success in such an environment inevitably relies on Students’ capability to actively and autonomously engage in the learning process (Wang et al., 2013), which is referred to as SRL (Zimmerman, 2008).

Specifically, SRL can be divided into three phases: forethought, performance and self-reflection (Zimmerman, 2000). In the forethought phase, L2 learners usually set goals and make strategic planning to achieve these goals. In the performance phase, L2 learners tend to employ certain strategies to monitor their performance and also self-control their learning process; in the last phase, learners self-evaluate their learning, report causal attributions, and adapt their performance systematically to achieve learning goals (Kitsantas, 2013; Viberg et al., 2020).

Abundant literature has pointed out that SRL skills are important for academic success (Dignath and Büttner, 2008), and may even impact subsequent “lifelong learning” (de la Harpe and Radloff, 2000). In a recent review exploring the association between SRL and MALL, the results demonstrated that m-learning could enhance learners’ SRL and learners’ SRL also contributed to m-learning (Palalas and Wark, 2020). In addition, some scholars have pointed out that SRL behavior was the most critical factor in predicting linguistic outcomes (Tseng et al., 2019). Other studies also found that self-directed learning helped learners achieve better and deeper learning outcomes (Dold, 2016; Yang, 2020).

Moreover, it has also been found that participants’ self-regulatory capacity (Fathi et al., 2018; Lei et al., 2022), learners’ learning efficiency and automaticity (Zhang et al., 2011) could be facilitated through MALL practices. Further, compared with traditional paper-based learning, learners tended to employ more self-regulated strategies in technology-based learning environments (Khezrlou and Sadeghi, 2012; Ma, 2017). Explanation for this can be the nature of mobile apps which created autonomy on learners and brought about a rise in self-regulation and learning management (Fathi et al., 2018); another justification can be the reachability and personalization of mobile Apps which enabled learners to use mobiles without restrictions of time and place (Fathi et al., 2018). To summarize, SRL and MALL are interdependent on each other.

The use of mobile technology for EFL learners, however, is not as often as one would expect in out-of-class MALL (Dashtestani, 2016; Botero et al., 2019). Zhang and Pérez-Paredes (2019), for instance, reported that learners were not regularly engaged in mobile language learning resources. Also, Nguyen and Takashi (2021) indicated that learners rarely use mobile devices for English learning outside the classroom. Though Luo (2019) found that participants used mobile Apps for language learning, the majority of them spent less than 20 min. Possible explanations for low mobile learning engagement range from lacking of confidence (Gamble et al., 2012), lacking of language learning partners (Lai and Zheng, 2018; Lai et al., 2018), afraid of getting incorrect feedback (Lai and Gu, 2011), to the disadvantages in App design (Luo, 2019).

Due to the obstacles in out-of-class mobile technology use, further research is much needed to investigate learners’ SRL behaviors and possible outside support that could facilitate EFL learners’ use of mobile technology in SRL beyond the classroom.

### Peer scaffolding

Though scaffolding was initially defined as the assistance given by a teacher/expert to a student/novice to reach a higher level of performance which may otherwise be impossible (Wood et al., 1976; Vygotsky, 1978), it was subsequently re-conceptualized to include the assistance shared among peers in collaborative learning (Crook, 1994; Donato, 1994; Bull et al., 1999). With the advent of modern technology, peer scaffolding could also be achieved online relying on educational software (Mortera-Gutiérrez, 2006; Mavrou et al., 2010), Internet resources (Bruce, 1997; Hughes, 2013) and communication tools (Järvelä et al., 2007; Xiao and Yang, 2019), which could help create a potentially powerful collaborative learning environment.

According to Winnips (2001), scaffolding could be divided into cognitive, metacognitive, and motivational scaffolds. While cognitive and metacognitive scaffolds provided assistance, prompts, hints, or suggestions regarding the content, resources, and strategies related to problem solving and learning management, motivational scaffolds included techniques designed to maintain or improve learners’ motivational state through encouragement or attribution (Azevedo et al., 2003).

#### Cognitive and metacognitive peer scaffolding

In online learning environment, as stated by Hannafin and Land (1997): “scaffolding is not solely limited to teacher-student or student-student interactions. Rather, technology-enhanced environments could also provide the conceptual scaffolding and means (resources, tools) to promote personal and individual reflection.” As for cognitive or metacognitive peer scaffolding achieved in online learning settings, previous research has mainly focused on the effect of peer scaffolding on problem solving (Brush and Saye, 2001; Kuo et al., 2014; Kim and Lim, 2019), reasoning (Chiu and Linn, 2014; Choi et al., 2015; Rashid et al., 2016), and online discussion activities (Lan, 2013; TechTarget, 2015; Liu, 2016; Hsieh, 2017; Yeh et al., 2017; Rasheed et al., 2021).

Among the three focused areas, online discussions are the most researched one. By using communicative tools like wikis, blogs, forums, chat, and so on, learners could discuss with peers, collaborate to finish a task (Wu et al., 2012; Nguyen, 2013), provide feedback, achieve the settlement of cognitive conflicts, articulation, and co-construction of knowledge (Crook, 1994; Luzón, 2006; Tsai, 2013). In online discussion groups, peer sharing activities such as resources sharing (e.g., video clips, graphics, notes.) (TechTarget, 2015; Hsieh, 2017) and learning experience sharing (e.g., vocabulary knowledge, the self-made concept maps, annotations, learning skills.) (Lan, 2013; Liu, 2016; Yeh et al., 2017; Rasheed et al., 2021) were also made easier. The use of online resources has been widely acknowledged for its benefits in helping language learners construct knowledge (Suthers, 2005; Hughes, 2013), and sharing learning experiences online could also help peer learners learn from each other (Kotturi et al., 2015).

#### Motivational peer scaffolding

Motivation refers to Students’ desire and willingness to make efforts toward and persist in the learning task (Schunk, 2008; Schunk et al., 2008), and could be influenced by Students’ judgments of their abilities to complete a task successfully and their perceptions of the possible benefits brought with task completion (Eccles et al., 1993; Wigfield and Eccles, 2000). Motivation was found to be correlated with behavioral, emotional, and cognitive engagement (Fredricks et al., 2004; Gagne, 2009; Reeve, 2013), such as actively engaging in academic activities, exhibiting interest, deploying strategies in understanding content, solving problems, and using information (Fredricks et al., 2004).

However, Alias (2012) has mentioned that “research on motivational scaffolding is relatively scare,” and more research needs to be done on the application of scaffolds to foster Students’ learning motivation in educational settings (Belland et al., 2013; Chen, 2014). Similarly, Chen (2014) emphasized the need for designing scaffolds that not only focused on Students’ cognitive status, but also psychological status that affected learning, fostered learners’ motivation while they acquired conceptual knowledge. Belland et al. (2013) argued for the necessity to incorporate motivational scaffolding to assist learners in maintaining motivation and interest, and has further proposed comprehensible guidelines for designing computer-based motivational scaffolds, which is adopted as the theoretical framework in the current study.

Six motivational goals are covered in this framework, among which promoting mastery goals, belonging, and expectancy for success could be well incorporated into motivational peer scaffolding design.

Firstly, mastery goals can help promote a broad range of positive outcomes, including intrinsic motivation, persistence, and deep processing (Hulleman et al., 2010), and are more supportive of group work than performance goals (Yamaguchi, 2001). Specifically, providing informational feedback and promoting cooperation are conducive to developing Students’ self-regulation and sense of connectedness (Reeve, 2009; Belland, 2013, 2014).

The second means to design motivational peer scaffolding is through promoting belonging. Belonging can be enhanced by encouraging shared goals and accommodating social goals. Shared goals should be based on group members’ ideas and interests (Tomasello et al., 2005); social goals refer to goals students pursue in social interactions with others (Wentzel, 1998; Dowson and McInerney, 2003). For instance, social responsibility goals may motivate students to work harder so as not to let their group down (Hijzen et al., 2007; Nelson and DeBacker, 2008).

In addition to promoting mastery goals and belonging, promoting expectancy for success is also an efficient means. Through enabling students to see a similarly abled peer can accomplish the task successfully without being overwhelmed (Schunk, 2003; Moos and Azevedo, 2009), peer modeling could be established.

Despite the existing established theoretical framework, nevertheless, previous literature focusing on motivational peer scaffolding in online learning settings is relatively scare, and most research focusing on motivational aspects of scaffolding has been achieved through the reminding, instant feedback and gamified functions embedded in learning Apps (Chen and Law, 2016; Berenji and Saeidi, 2017; Kohnke, 2020) or have been exerted by teachers (Mackiewicz and Thompson, 2013; Duffy and Azevedo, 2015). There, exists, however, notable efforts at providing motivational scaffolding exerted by peers in the work of Kim et al. (2006), Tuckman (2007), and Mi et al. (2020). Tuckman (2007), for instance, designed peer-to-peer or instructor-to-peer online support group meetings and found that procrastinating students performed better with this type of motivational scaffolding; Kim et al. (2006) worked on a virtual peer who was responsive to Students’ affective status, and also played a modeling role in the learning process. Mi et al. (2020), on the other hand, mentioned that peer scaffolding in terms of achievements sharing in online learning groups could enhance Students’ learning interest and motivation, yet no experiment was carried out to confirm this self-report survey finding.

Considering that in online learning settings, peer learning has long been identified as an important learning strategy to foster social interaction and better engagement which can hardly be facilitated by a single instructor (Burke, 2011; Rasheed et al., 2021), whether motivational scaffolding exerted by peers could equally support mobile learning as cognitive or metacognitive ones warrants further attention.

### Interaction between self-regulated learning and peer scaffolding

Previous research has shown that communication tools could facilitate help-seeking, self-monitoring by receiving feedback from peers and elaborating on their own understandings through online discussions (Nicol, 2009; Kitsantas and Dabbagh, 2010, 2011; Dabbagh and Kitsantas, 2012). In addition, the more goal-oriented students were, the more likely they were to interact with peers (Yang and Zhang, 2013), which was important in determining Students’ successful learning experiences (Cho and Jonassen, 2009; Garner and Bol, 2011).

Specifically, when it comes to language learning, it has been pointed out that peer scaffolding delivered via the Telegram app could help students learn the intended vocabulary items better compared with the traditional vocabulary learning approach (Ghobadi and Taki, 2018; Heidari Tabrizi and Onvani, 2018; Mansouri and Mashhadi Heidar, 2019); flipped classrooms incorporating peer feedback could motivate the class to be more interactive (Crouch and Mazur, 2001; Chen Hsieh et al., 2017; Hung, 2017). Moreover, formative assessment activities involving peer and teacher feedback practices were also found to develop learners’ self-regulation capability (Clark, 2012; Xiao and Yang, 2019).

Taken together, current research mainly has three limitations: firstly, consensus has not been reached as to the effect of mobile-assisted vocabulary learning; secondly, research on learners’ SRL behaviors in out-of-class MALL context is not yet sufficient; thirdly, whether motivational peer scaffolding in online learning settings could equally scaffold learners’ self-regulation just as cognitive and meta-cognitive scaffolding warrants further exploration. This study, by examining the effects of self-regulation and motivational peer scaffolding on mobile-assisted vocabulary learning, aims to address the following questions:

Q1: What’s the effect of self-regulation on mobile-assisted vocabulary learning?

Q2: What’s the effect of peer scaffolding on mobile-assisted vocabulary learning?

Q3: To what extent does self-regulation mediate the effect of peer scaffolding on mobile-assisted vocabulary learning?

## Methodology

Based on previous studies in SLA and MALL, the current research aims to investigate the effects of peer scaffolding and self-regulation on mobile-assisted vocabulary learning.

### Shanbay App

Shanbay App is a popular vocabulary learning App used on mobile phones or computers, and was chosen to measure participants’ vocabulary learning achievements. The definitions and example sentences provided in the App are adopted from the Collins Advanced Learner Dictionary.

It’s innovative in autonomously setting a learning goal for users based on their own needs and proficiency levels. Besides, the App could also create a certain number of target words for users to do self-assessments or quizzes (Zou et al., 2018). The self-assessment mode mainly tests learners’ familiarity with learned words; the quiz mode includes word translation, word spelling, and word listening activities. Furthermore, interaction opportunities are also provided in that learners can form learning groups, communicate with other users in the forum, and share their daily learning achievements to other social platforms. Moreover, the App also contains a motivating mechanism by awarding users with medals which could be used to buy some other vocabulary books (Zou et al., 2018).

### Participants

This study focused on the population of Chinese higher education EFL learners in a comprehensive university. Altogether 71 students aged between 17 and 19 from two intact parallel classes were chosen as participants. All of them were enrolled in the *College English Advanced Course* and have passed the CET-4 examination (Mean = 588.17, SD = 39.00), and are intermediate-level English language learners. The two classes were randomly assigned as two groups: the experimental group with peer scaffolding (37 people) and the control group without peer scaffolding (34 people). Participants in each group were further grouped into small vocabulary learning teams consisting of 4–5 teammates, based on the statement that peer learning usually takes place by forming small groups consisting of 3–5 members (Agrawal et al., 2014; Esfandiari et al., 2019).

In the experimental group, participants were required to share their daily vocabulary learning achievements with their peers in online learning groups outside the classroom. Participants in the control group, however, were not asked to share their daily vocabulary learning gains with peers, but were required to upload their vocabulary learning achievements regularly to the researcher.

### Instruments

A mixed method was used in the current study to provide a more comprehensive understanding of the studied phenomenon (Creswell, 2014). In the current study, both qualitative data (semi-structured interview data) and quantitative data (log data from Shanbay App and questionnaire data) were collected.

#### Surveys

The Online Self-regulated Learning Questionnaire (OSLQ) (Barnard et al., 2008) was adopted in the current study, which was designed specifically to incorporate contextual differences in online learning settings (Broadbent, 2017). It is a short form of Zimmerman’s work to reflect a multi-dimensional conception of SRL (internal consistency α = 0.93), contains 24 items covering six important categories of self-regulation: (a) environment structuring; (b) goal setting; (c) time management; (d) help seeking; (e) task strategies; (f) and self-evaluation.

Specifically in our experiment, to measure Students’ self-regulation in online vocabulary learning settings, an adapted form of OSLQ (Barnard et al., 2008) was employed (see Supplementary Appendix I). The modified questionnaire contains two parts. In Part 1, participants’ demographic information (gender, age, student ID) is collected. Part 2 consists of 23 items (one was deleted) that covers the initial six categories, and participants had to indicate the degree to which each item was characteristic or true of them using a 5-point Likert scale from strongly disagree (1) to strongly agree (5). The deletion was made to make the questionnaire more appropriate in this mobile-assisted vocabulary learning context.

Considering the participants’ linguistic background, the questionnaire was delivered in Chinese to make the results more reliable. To ensure the reliability of the measurement, a pilot study was conducted by delivering the survey to 49 participants, the results of which showed good internal consistency (Barnard et al., 2008) with Cronbach’s Alpha = 0.864. In terms of validity, the researcher, English experts and teachers have examined the survey contents, and revised some items to secure the expert validity. Besides, confirmatory factor analysis was also performed, with X^2^/df < 3, RMSEA < 0.10, RMR < 0.05, CFI > 0.911.

#### Semi-structured interviews

Semi-structured interviews were adopted to further examine Students’ mobile vocabulary learning experience as well as the possible effects of self-regulation and peer scaffolding in the learning process. Both online individual interviews through text chats and face-to face individual interviews were incorporated in the current study. In particular, participants were asked about their SRL behaviors, the possible role of peer scaffolding in the learning process and their mobile vocabulary learning experience with Shanbay App. The specific interview questions can be found in Supplementary Appendix II. To achieve more extensive responses from the participants, the order of questions were not fixed and some questions were probed further (Ahmad et al., 2017).

Altogether 22 participants were interviewed individually in the current experiment, and in each group, those who performed above the average, about the average and below the average were selected based on the mean of their vocabulary learning achievements (days spent in learning and sum of learned words). The descriptive statistics of the interview data was shown in Table 1. The interviews were tape recorded and later transcribed into English scripts as they were initially conducted in Chinese to make the data more reliable. A smaller pilot study was also conducted with four students before the formal interviews to validate the interview questions.

**TABLE 1:** Descriptive statistics of interview data.

	Number	Average time
Online individual interviews	5	24.87 min
Face-to-face individual interviews	17	17.92 min

### Research procedure

**FIGURE 1:**
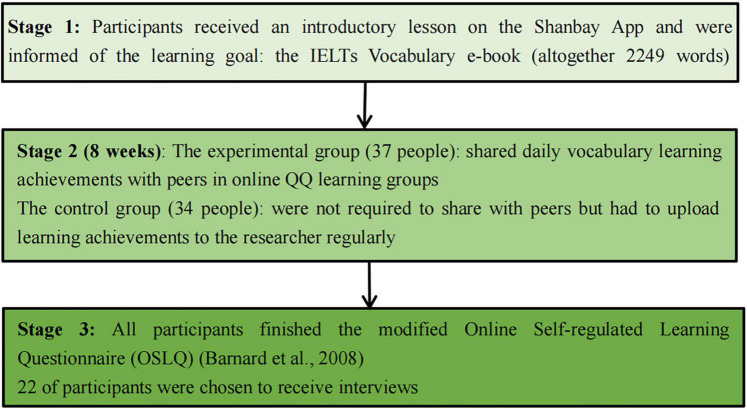
Research procedure of the experiment.

Research procedure of the experiment is displayed in Figure 1.

### Data analysis

As for the analysis of quantitative questionnaire data, participant’ responses were coded as scores manually. The total score of each item is 5, which is equivalent to participants’ responses to that item ranging from 1 (strongly disagree) to 5 (strongly agree). Participants’ scores in OSLQ were also computed and those with scores above the mean were identified as high self-regulated learners and those below the mean were low self-regulated learners. Therefore, there were two groups (peer scaffolding group and the control group) within which there were both high and low self-regulated learners.

To analyze the log data from Shanbay App, several steps have to be followed. Firstly, the number of days spent in vocabulary learning and the total number of learned words were manually calculated by the researcher. Then, to ensure that the data is suitable for performing the next tests, the normality of distribution and homogeneity of variance assumptions were examined. An independent-samples *t*-test was conducted twice to see if the two groups differed on mobile vocabulary learning results.

**FIGURE 2:**
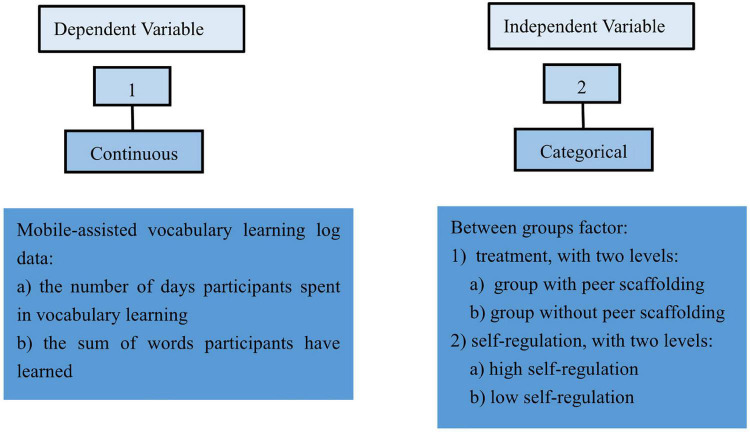
Two-way between-groups ANOVA.

To answer the research questions, it was initially required to make sure that the experimental group and the control group did not differ significantly from each other in terms of self-regulation. An independent-samples *t*-test was run on survey scores of the two groups. Then, a factorial ANOVA was adopted (see Figure 2). The independent variable includes two between-group variables: peer scaffolding and self-regulation; the dependent variable involves the number of days participants spent in vocabulary learning and the sum of words they have learned. An interaction effect between the two independent variables has also been added in data analysis.

Then, the qualitative interview data was analyzed following three steps—description, analysis and interpretation (Wolcott, 1994). Firstly, the initial Chinese transcript was translated into English. Then, an inductive and comparative strategy was employed to group the data into different themes, patterns or categories (Merriam and Tisdell, 2015). Finally, the interpretation stage was performed to confirm the meaning of the data, and triangulation methodology was further conducted to establish the validity and credibility of the research to find out if conclusions from each source are the same (Guion et al., 2011). During data analysis, the primary focus has been on the data that would support or refute the results of the quantitative data analysis, thus making the studies’ answers more complete and comprehensive. Besides, the interview data was also expected to shed light on the explanation of the research results.

## Results

This study aims to explore the effects of self-regulation and peer scaffolding on mobile-assisted vocabulary learning. Participants’ learning results are shown in Table 2. An independent-samples *t*-test showed that the experimental group and the control group differed significantly in learning results in terms of the number of days spent in vocabulary learning (*t* = 4.78, *p* = 0.000, df = 46.15, 95% CI = 5.36, 13.17), and the sum of words participants have learned (*t* = 2.18, *p* = 0.033, df = 69, 95% CI = 30.13, 674.06) (see Table 3).

**TABLE 2:** Participants’ vocabulary learning log data.

	Number of days			Sum of words		
		
	Mean	SD	[95% CI]	Mean	SD	[95% CI]
Experimental group (*N* = 37)	65.00	4.86	[63.38, 66.62]	2430.27	587.29	[2234.46, 2626.08]
Control group (*N* = 34)	55.74	10.30	[52.14, 59.33]	2078.18	767.28	[1810.46, 2345.89]

**TABLE 3:** Output from the independent samples *t*-test.

*t*-test for equality of means							

	*t*	df	Sig.		Mean difference	Std. error difference	95% CI of the difference
Number of days	4.78	46.15	0.000	9.26	1.94	5.36	13.17
Sum of words	2.18	69	0.033	352.09	161.39	30.13	674.06

### Effect of self-regulation on mobile-assisted vocabulary learning

As revealed by the survey, the mean score is 83.72 (total score = 115), with 42 students performing above the mean and 29 below the mean across the two groups, who were identified as high self-regulated and low-regulated learners, respectively.

The factorial 2-way ANOVA tests showed that self-regulation did not have a statistical effect on the number of days spent in vocabulary learning (*p* = 0.87) (Table 4); whereas a main effect of self-regulation on the sum of learned words [*F*_(1, 67)_ = 50.268, *p* = 0.000, partial eta-squared = 0.43] has been observed (Table 5). The effect size showed that this factor accounted for *R*^2^ = 43% variance in the data, which was a medium effect.

**TABLE 4:** Factorial ANOVA output: Effects of self-regulation and peer scaffolding on the number of days.

Source	Type II SS	df	Mean square	*F*	Sig.	Partial eta squared
Corrected model	1599.558^a^	3	533.186	8.362	0.000	0.272
Intercept	260422.535	1	260422.535	4084.432	0.000	0.984
Self-regulation	1.730	1	1.730	0.027	0.870	0.000
Peer scaffolding	1511.697	1	1511.697	23.709	0.000	0.261
Self-regulation*peer scaffolding	76.981	1	76.981	1.207	0.276	0.018
Error	4271.906	67	63.760			
Total	266294.000	71				
Corrected total	5871.465	70				

^a^R Squared = 0.272 (Adjusted R Squared = 0.240).

**TABLE 5:** Factorial ANOVA output: Effects of self-regulation and peer scaffolding on the sum of learned words.

Source	Type II SS	df	Mean square	*F*	Sig.	Partial eta squared
Corrected model	16471478.704^a^	3	5490492.901	20.937	0.000	0.484
Intercept	363173156.113	1	363173156.13	1384.919	0.000	0.954
Self-regulation	13182074.102	1	13182074.102	50.268	0.000	0.429
Peer scaffolding	1677973.714	1	1677973.714	6.399	0.014	0.087
Self-regulation*peer scaffolding	1092864.953	1	1092864.953	4.168	0.045	0.059
Error	17569689.183	67	262234.167			
Total	397214324.000	71				
Corrected total	34041167.887	70				

^a^R Squared = 0.484 (Adjusted R Squared = 0.461).

The qualitative interview data provided extra support for the positive effect of self-regulation on mobile vocabulary learning performance. In general, participants’ SRL behaviors could be summarized from goal setting, time selecting, employment of learning strategies (note taking, summarizing, memorization enhancement methods.) and help seeking.

As for goal setting, most participants indicated that they adhered to short-term goals set autonomously by Shanbay App. For those who tended to set a relatively higher learning goal, they performed better in vocabulary learning accordingly. Participant 20, for example, mentioned that “I set a goal at 100 words a day, though I cannot remember all of them, the number of words I have learned is still large,” and it follows that he learned several times the sum of words than his peers.

In addition, time selecting also influenced mobile vocabulary learning performance. Compared with learning at fragmentary time, fixed periods of time could help students form learning habits and better persist in the mobile learning process. As indicated by participant 15: “I will choose a fixed time to learn, in the beginning at noon, and later I get accustomed to learning at around 11 p.m. at night.” Participant 21 added: “I also prefer fixed time.learning at fragmentary time will make me very busy without actually learning anything!”

Learning at fragmentary time, in contrast, had its own drawbacks. As indicated by participant 17: “At the very beginning I spend 15–20 min learning new words, but now I only spend around 10 min in swiftly skimming over them.” That said, deep learning of new words may be hard to achieve. In addition, participants also indicated that in fragmentary time they tended to review words rather than learning new words, as the efficiency of learning usually cannot be guaranteed in this way (participants 3, 5).

In addition to goal setting and time selecting, individualized SRL strategies could also affect mobile vocabulary learning. For students who have explored more diverse functions of the App like word testing, peer learning and note-sharing function, they tended to persisted well and performed relatively better than their peers. While word testing function could help establish the form-meaning connection of words, thus helped memorization (participants 1, 2, 6, 14), peer learning function could remind them to persist in the learning process (participants 6, 14, 19). But for those who performed relatively worse than their peers, they usually did not explore much of Shanbay’s function, and gradually gave up learning (participants 16, 21).

Note taking and summarizing was another typical learning behavior displayed by self-regulated participants, “Sometimes I will take notes, and add them to the words encountered in reading tests, and review them in my free time” (participant 4). Similarly, participants 5, 14, and 15 also indicated that they would note down words similar in spelling. It follows that note taking was conducive to vocabulary learning, as noted by participant 3: “I would note down words similar in meanings together with those I met in reading tests, and it helps me in composition writing with more vocabulary choices.”

Specifically, participant 20 who learned much more than his peers shared his SRL strategy: “I once learned from a teacher that you should spend less time on a single word and repeat learning it, then within the same period, you will encounter one word for several times. That’s why I learned so many words. And it’s not that you have skimmed over 200 words and acquired all of them, but even if you only remember 50% percent, the amount is still large.”

When asked about whether they would consult or communicate with their peers in the learning process, the majority of them indicated they mainly learned by themselves. However, it’s interesting to note that some students in the control group (without peer scaffolding) established learning groups of their own, shared with their peers daily learning achievements, and also discussed about their learning progress (participants 7, 10, 12, 13). Such a SRL behavior was conducive to vocabulary learning in that students tended to persist better with peers’ sharing and supervision.

Besides the increasing vocabulary size, the facilitating role of Shanbay App was also manifested in reading comprehension tests, especially for those better persisted in using the App. “I think it helps me a lot, for sometimes when I do reading tests and suddenly encounter a newly learned word, I will have a sense of achievement. And I feel that there are fewer unfamiliar words in the reading tests (participants 1, 3).” With the decreasing number of unfamiliar words, reading speed has also greatly improved (participants 5, 10). As for whether they would like to continue using the App, the majority of students indicated that they would still use the App to learn other vocabulary books like CET-6 in the future.

### Effect of peer scaffolding on mobile-assisted vocabulary learning

To explore the effect of peer scaffolding on mobile-assisted vocabulary learning, participants in the experimental group and the control group should not differ significantly in terms of self-regulation scores, which has been tested by an independent samples *t*-test, with *p* = 0.673, which indicated that there were no significant differences in self-regulation between the two groups.

As revealed by the results of the factorial ANOVA test (see Table 4), the main effect of peer scaffolding on the number of days spent on vocabulary learning was statistical [*F*_(1, 67)_ = 23.709, *p* = 0.000, partial-eta squared = 0.261], and peer scaffolding could explain 26.1% of variance in the data, which was a medium effect. A statistical effect of peer scaffolding on the sum of words has also been found, with [*F*_(1, 67)_ = 6.399, *p* = 0.014, partial-eta squared = 0.087], which means that peer scaffolding could explain 8, 7% of variance in the data, which was a small effect.

The qualitative interview data further confirmed the effect of peer scaffolding on mobile-assisted vocabulary learning results. For participants in the experimental group, the majority of them indicated that their peers’ sharing could serve as a reminding function: “Sometimes I forget learning, and others’ sharing would remind me of this” (participants 1, 2, 4, 6). Besides, some of them also discussed with their peers about their learning progress in online learning groups: “Sometimes we will discuss about our recent progress in vocabulary learning and maybe some problems we have encountered (participants 8, 12). Another participant also indicated that: “Group discussions took place occasionally, and mostly revolved around the difficulties in learning process, like a complex word that could hardly be remembered” (participant 13). However, as for whether they would be motivated to set a higher daily learning goal, most of them indicated that they just followed their own pace independent of others’ influence. There were only few students who thought that others’ sharing would motivate them to learn more words: “I don’t want to lag behind them, so I reset my learning goal from 80 to 120” (participant 3).

As for participants in the control group, it’s interesting to note that peer scaffolding still plays a role for some of them. Several participants indicated that they have established their own learning groups and shared with each other their daily learning achievements. “We have our own groups and if my peers learned more than me, I will be motivated to learn more but within the proper range, and I think this is a mutual scaffolding process” (participant 13); “We share with classmates, and I think peers’ sharing play a role in reminding and supervising” (participants 7, 10, 12).

### The interaction effect of self-regulation and peer scaffolding on mobile-assisted vocabulary learning

**FIGURE 3:**
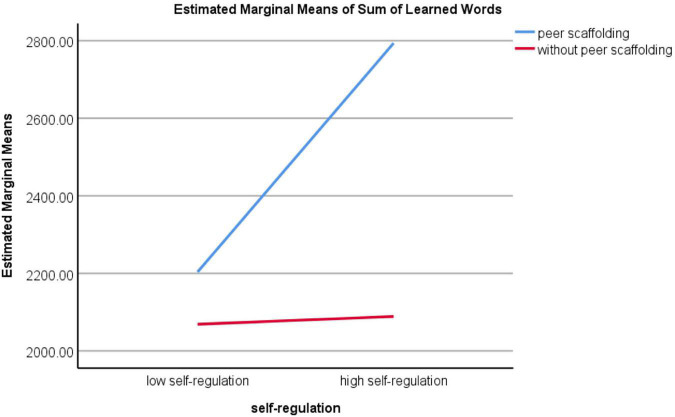
The interaction effect of self-regulation and peer scaffolding on the sum of learned words.

It was found that there was an interaction effect of self-regulation and peer scaffolding on the sum of words participants have learned (Table 5), with [*F*_(1, 67)_ = 4.168, *p* = 0.045, partial-eta squared = 0.059]. However, when the dependent variable is the number of days spent in vocabulary learning, no interaction effect has been found (see Table 4), with *p* = 0.276. The interaction effect was further confirmed in Figure 3. As shown in the line chart, when peer scaffolding was not imposed on students, no significant difference has been found concerning the sum of learned words between low self-regulated and high self-regulated students. However, when peer scaffolding was provided to them, high self-regulated students tended to perform better than low self-regulated students.

This result could also be explained by the interview data. For participants in the experimental group, peer scaffolding could motivate students to persist in the learning process (participants 1, 2, 4, 6), which is a manifestation of SRL behaviors. Participant 1, who reported high scores in self-regulation survey, mentioned that: “There is a reminding function of sharing with others sometimes I forgot learning, and others’ sharing would remind me of this.” Besides, some participants in the control group were also scaffolded by their peers by forming learning groups of their own (participants 7, 10, 12, 13). These participants actively engaged in help seeking and peer communication activities, which was also a demonstration of high self-regulating capabilities.

In addition to peer scaffolding achieved through different treatments, it could also be achieved through the App itself. For those highly self-regulated students, they were more likely to explore various functions of the Shanbay App, among which peer learning function could scaffold their SRL process: “Peer learning function helps me to some extent, as sometimes I see my peer learn in the morning, and I will think about complete learning earlier “ (participant 6).

In contrast, however, as for some students who reported low scores in self-regulation survey, they demonstrated their indifference to others’ sharing and mentioned that they would not be scaffolded by others, and only learned if they had time (participants 16, 21).

## Discussion

This study aims to find the effects of self-regulation and peer scaffolding on learners’ mobile-assisted vocabulary learning performance. Based on the results, it could be confirmed that mobile-assisted vocabulary learning was beneficial to students in that most students could persist well within the experimental time and almost finish the required learning tasks. Besides, compared with paper vocabulary books, this way of learning was more user-friendly in that it could allow learners to make their personalized goals, learn anytime and anywhere and also interact with peers.

### The effect of self-regulation on mobile-assisted vocabulary learning

An important finding in our research revealed by the survey was that the participants in general were relatively highly self-regulated with an average score for each item over 3.5, which makes it easier to carry out research in online autonomous learning environment.

It has been found that self-regulation had a significant effect on the sum of words participants have learned. This result was in line with previous research in which SRL was associated with Students’ achievements in online learning environments (Viberg and Andersson, 2019). Similarly, Tseng et al. (2019) have also found that SRL behavior was one important factor in predicting linguistic outcomes, and more self-regulated students usually had more preferable learning outcomes (Rashid and Asghar, 2016; Broadbent, 2017).

To be specific, as revealed by the research results, participants’ SRL behaviors could be summarized from goal setting, time selecting, employment of individualized learning strategies and help seeking. Notably, those who demonstrated more SRL behaviors like choosing fixed time to learn, exploring the diverse functions of Shanbay App, employing memorization tactics, taking notes and summarizing regularly were more likely to perform better in mobile vocabulary learning outcomes. This result, however, partly conflicts with Viberg and Andersson (2019)’s research in which time management and help-seeking were rated particularly highly for those self-regulated participants. In our research, only few students would consult their peers in the learning process, and most of them indicated that this was a SRL process without the need to seek others’ help. This may be ascribed to the different design of learning Apps. In Viberg and Andersson (2019)’s research, an immediate feedback function was incorporated into the App, which made it easier for participants to interact with peers or instructors online. In our research, however, peers cannot see each other’s learning progress in Shanbay App directly, and also cannot provide feedback in this App. Another reason may be that besides vocabulary learning, grammar, reading, and listening learning tasks were also incorporated into the App design in Viberg and Andersson (2019)’s research, during which process peers’ help may play a greater role than in vocabulary learning alone.

However, no positive effect of self-regulation on the number of days spent on vocabulary learning has been found. This may be explained by the duration of our experiment. As our research only lasts for around 2 months, the participants did not differ significantly from each other in terms of the days spent on vocabulary learning, especially for the experimental group who were motivated to persist well in the learning process. Another reason may be that the questionnaire used in our study did not measure participants’ persistence in learning but rather measured their specific SRL behaviors displayed in the learning process. As such, items measuring Students’ persistence in learning could be added in future research. In addition, follow-up study is also needed to differentiate students from each other in terms of the days spent in persistent vocabulary learning.

In technology-enhanced teaching and learning environments, the combination of mobile devices with traditional classroom-based teaching could help students to autonomously engage in the SRL process. In addition, training of self-regulation skills could also be provided by teachers to enable students become more self-regulated EFL learners.

### The effect of peer scaffolding on mobile-assisted vocabulary learning

In our research, both quantitative and qualitative findings revealed that peer scaffolding did play some role in improving Students’ vocabulary learning performance. A main effect of peer scaffolding on mobile vocabulary learning results has been observed on both the number of days and sum of words; the interview data also revealed the facilitating role of peer scaffolding in participants’ vocabulary learning process, which coincides with Mansouri and Mashhadi Heidar’s (2019) research in which peer scaffolding delivered via a technology-enhanced group significantly affected vocabulary learning.

The findings of the current study are also in accordance with the findings of investigations exploring the effects of using other learning Apps on vocabulary learning, such as the positive effect of using Telegram on vocabulary learning (Ghobadi and Taki, 2018; Heidari Tabrizi and Onvani, 2018). One justification of these results maybe the accessibility and user-friendliness of online learning Apps, and also the motivational aspects of using technology for language learning achieved through online peer scaffolding. In line with Tuckman (2007)’s finding, this study also found the facilitating role of motivational peer scaffolding in enhancing Students’ learning motivation, which further translated into better academic performance. Nevertheless, his research only focused on procrastinating students whereas our research has paid attention to both low self-regulated and high self-regulated students, and found that high self-regulated students were more likely to be scaffolded by peers. Besides, the research results also extend Mi et al. (2020)’s claim that achievement sharing among peer learning groups could motivate students to learn. The experiment conducted in our research confirms that learners could be motivated by peers to better persist in vocabulary learning, spent more time in online SRL and also acquired more words.

In addition, the results may also be attributable to the “audience effect,” as in line with Regan and Zuern (2000)’s research which found that participants would compose articles with better quality and quantity with online audience; similarly, Lan (2013) has also noted the effect of co-sharing on L2 learners’ vocabulary learning strategy (VLS) construction, and those who constructed more VLSs through co-sharing outperformed those who did not use this function. In the current study, participants in the experimental group were required to share their daily vocabulary learning results with their peers in small learning groups, during which the “audience effect” may exert certain effects. With peers’ sharing and supervision, students tended to perform better in vocabulary learning.

The finding also suggests that there is a need to go beyond just providing peer sharing as the sole means of achieving motivational peer scaffolding, as most students interviewed indicated that they would not be motivated to learn more words. Instead, new strategies such as self-regulation training of participants could be explored, along with MALL integration with expected learning activities like between-group competitions to foster the cooperation and sense of belonging among group members, which may be conducive to improved mobile vocabulary learning performance.

The facilitating role of peer scaffolding in mobile-assisted vocabulary learning also sheds light on the improvement of teaching methods in technology-enhanced learning settings. By grouping students into smaller online learning groups, they are supposed to scaffold and motivate each other to be more actively engaged in the out-of-class SRL process, which may further translate into improved academic performance.

### The interaction effect of self-regulation and peer scaffolding on mobile-assisted vocabulary learning

An interaction effect has been observed between self-regulation and peer scaffolding on the sum of words participants have learned. As revealed by the interaction plot, as for low self-regulated learners, the exertion of peer scaffolding did not greatly improve their learning performance, yet when it comes to high self-regulated learners, the facilitating role of peer scaffolding was quite obvious. As such, the effect of peer scaffolding on mobile vocabulary learning has been influenced by self-regulation.

In the current experiment, though most participants indicated that they would not be motivated to learn more words under peers’ influence, nearly all of them agreed that peer sharing in groups served as a reminding function, which helped them persist in the learning process. It follows that the increasing number of days spent in vocabulary learning would inevitably translate into increasing sum of words, considering that they all adhered to the fixed daily learning goals set autonomously by the App. The results could also be explained by the qualitative interview data, which revealed that participants with better vocabulary performance usually manifested more diversified SRL behaviors, and the reminding and supervising function of peer scaffolding did help them persist in the learning process. Yet for those participants with worse vocabulary learning performance, they may not care much about others’ sharing and just followed their own learning pace if they had time.

Besides, the findings of this study are also in line with previous research which observed the facilitating role of peer scaffolding in self-regulation enhancement. Dabbagh and Kitsantas (2012), for instance, mentioned that social and collaborative activities in online learning communities could help students engage in the self-regulation process of goal setting, self-monitoring, and help-seeking. Further, other self-regulatory skills like time-management and task strategies could also be fostered through learning technologies which helped students engage in conversational interactions with peer learners (Kitsantas, 2013).

By creating informal online learning groups, a supportive learning environment could be established. Through interaction with peers and experts, Students’ motivational beliefs and task interests could be promoted (Kitsantas and Dabbagh, 2010), which was conducive to Students’ SRL process (Kitsantas and Dabbagh, 2011). Further, Nicol (2009) also pointed out that peer scaffolding achieved through online discussion groups helped encourage students to take control of their own learning, resulting in higher levels of satisfaction and learning gains.

## Conclusion

The study investigates the effects of self-regulation and motivational peer scaffolding on mobile-assisted vocabulary learning. Results showed that peer scaffolding helped participants better persist in the mobile vocabulary learning performance with more days spent in learning and more acquired words; the main effect of self-regulation and interaction effect of self-regulation and peer scaffolding have also been observed on the sum of words participants have altogether learned within the experimental time.

The results of the study have also some insightful pedagogical implications for EFL instructors and learners. As for teachers, they can integrate mobile learning Apps like Shanbay into their traditional classroom-based teaching. For some language skills like vocabulary, teachers may not have enough time to teach in class due to restricted instructional time. In this sense, they can take full use of mobile Apps beyond the classroom to assist teaching and also add some varieties and innovations to traditional vocabulary teaching and learning (Lei, 2017; Fathi et al., 2018), which also follows the requirements of the Ministry of Education that EFL teaching in higher educations should make full use of modern information technology and devices to make English teaching and learning more personalized and autonomous (General Office of the MOE, 2007). Specifically, instructors could provide students with self-regulation skills training and group them into smaller learning groups to help them better persist in the mobile learning process. As to learners, with the assistance of the personalization and motivation aspects embedded in mobile Apps, they could learn autonomously and better engage in self-regulated mobile learning process (Fathi et al., 2018).

However, the generalization of the results is limited due to the small sample size, the usage of one specific mobile learning App and the specific context of participants. Besides, the learning tasks are set by the researcher, and Students’ personalized learning goals should also be considered. Further, demographic variables such as prior mobile language learning experience could also be taken into account (Huang and Yu, 2019). Considering that the experimental time is limited to differentiate students in terms of the number of days spent on vocabulary learning, follow-up study is warranted to keep track of participants’ SRL behaviors after the formal experiment. Finally, more diversified motivational peer scaffolding activities could also be incorporated to enhance peer cooperation in future research.

## Data availability statement

The data analyzed in this study is subject to the following licenses/restrictions: The dataset will be available upon request by contacting the corresponding author. Requests to access these datasets should be directed to YZ, zyh_980428@163.com.

## Ethics statement

Ethical review and approval was not required for the study on human participants in accordance with the local legislation and institutional requirements. Written informed consent to participate in this study was provided by the participants’ legal guardian/next of kin.

## Author contributions

All authors listed have made a substantial, direct, and intellectual contribution to the work, and approved it for publication.
