# Supplementary material for: Mobile-assisted vocabulary learning through the Shanbay App outside the classroom: Effects of self-regulation and peer scaffolding
Source: Front Psychol. 2022 Oct 6;13:993224. doi: 10.3389/fpsyg.2022.993224 (PMC9583384; doi:10.3389/fpsyg.2022.993224)
Supplement: Supplementary file 1 [file Table_1.DOCX]

Supplementary Material

# Supplementary Tables

**Appendix I.** Online self-regulated learning questionnaire.

| **Item** | **Subscale** |
| --- | --- |
| I set goals to manage vocabulary learning time. | Goal Setting |
| I set certain standards for my daily vocabulary learning, for example, the specific number of new words to be learned. | Goal Setting |
| I set short-term goals (daily or weekly) for vocabulary learning. | Goal Setting |
| I set long-term goals (monthly or for the semester) for vocabulary learning. | Goal Setting |
| I don’t compromise the quality of vocabulary learning just because it is an online self-regulatory learning process. | Goal Setting |
| I choose the location where I study to avoid too much distraction. | Environment Structuring |
| I find a comfortable place to learn vocabulary. | Environment Structuring |
| I know where I can study most efficiently for vocabulary learning. | Environment Structuring |
| I choose a time with few distractions for vocabulary learning. | Environment Structuring |
| I try to make thorough notes when learning vocabulary through the Shanbay App, as it’s more important for learning online than in a regular classroom. | Task Strategies |
| I read vocabulary loudly to fight against distractions and enhance memorization. | Task Strategies |
| I often share my vocabulary learning outcomes with my classmates through social media (e.g., QQ or Wechat). | Task Strategies |
| Besides Shanbay App, I resort to other sources to learning vocabulary such as dictionaries, paper vocabulary books and so on. | Task Strategies |
| I allocate extra time for vocabulary learning because I know it’s time-demanding. | Time Management |
| I try to schedule the same time every day to study for vocabulary. | Time Management |
| Although I don’t have to attend the *College English Advanced Course* every day, I still try to distribute my vocabulary learning time evenly across days. | Time Management |
| I find someone who is knowledgeable in English vocabulary so that I can consult with him or her when I need help. | Help Seeking |
| I share my problems in vocabulary learning with my classmates online so we know what we are struggling with and how to solve problems. | Help Seeking |
| If needed, I try to discuss with my classmates face-to-face on English vocabulary learning. | Help Seeking |
| When I encounter a problem in vocabulary learning, I would like to get help from the teacher or the teacher assistant online. | Help Seeking |
| I summarize my vocabulary learning to examine my understanding of what I have learned. | Self-evaluation |
| I communicate with my classmates to find out how I am doing in my vocabulary learning. | Self-evaluation |
| I communicate with my classmates to find out what I am learning that is different from what they are learning. | Self-evaluation |

**Appendix II.**

| **Interview Questions** |
| --- |
| 1. During this period of mobile learning, which one do you prefer: traditional paper word books or mobile word learning Apps? And could you provide the reasons? |
| 1. Some of you reflected that they clicked quickly using this App yet remembered only few words; in some cases they could just recognize the words but could not spell them out. Have you encountered the same problems? |
| 1. Would you employ other functions of Shanbay to enhance memorization? For instance, “word testing” function, “peer learning” function, “note-taking” function and so on? Or would you combine mobile learning with traditional learning using paper word books? |
| 1. What do you think of the learning efficiency of “3+1 learned words+new words”learning mode provided by Shanbay? Do you feel that the amount of revision is a bit heavy and would it affect the subsequent learning of new words**?** |
| 1. Would you share your daily vocabulary learning achievements with peers in online learning groups or other social platforms (e.g., QQ, Wechat, Weibo...)? |
| 1. Would your peers’ sharing scaffold your own vocabulary learning? For instance, if you see that your classmates have learned more words than you and have persisted in learning for longer time, would you think about learning more? |
| 1. In addition to the goal set autonomously by Shanbay App, would you set a certain goal for yourself? If yes, do you prefer short-term goals (e.g., on a weekly basis) or long-term goals (on a monthly or semester basis)? |
| 1. Do you usually choose fixed periods of time or fragmentary time (e.g., buying lunch, waiting for the lift, etc.) to study words? And if you prefer fragmentary time, would the environment interfere with your learning ? |
| 1. Would you take some hand-written notes in learning with Shanbay? And would you review your notes? |
| 1. Would you summarize the words you have learned for a period of time? |
| 1. When you encounter problems in vocabulary learning, would you consult your peers or teachers, or prefer to solve by yourself like resorting to the dictionary? |
| 1. Would you discuss with your peers about your vocabulary learning progress? |
| 1. After this period of study, do you think that using Shanbay to learn words is conducive to your English study? For example, is it easier to do reading comprehension tests, or do you have more vocabulary to choose from in composition writing tasks? |
| 1. Do you think that Shanbay has some shortcomings in its design? Like sometimes you click fast and pass a word you do not memorize, and cannot find it out easily? |
| 1. Have you used other word learning Apps (e.g. Baicizhan, Momo...)? Compared with Shanbay, which one do you prefer? What’s the advantages or disadvantages of these Apps? |
| 1. Will you insist on using Shanbay for vocabulary learning in your future study life, or prefer other word learning Apps? If you prefer paper word books, what’s the reasons ? |
